# 220. Acute kidney injury incidence comparison of vancomycin trough-based vs AUC/MIC monitoring at a tertiary care hospital

**DOI:** 10.1093/ofid/ofac492.298

**Published:** 2022-12-15

**Authors:** Rafael E Ruiz Gaviria, Sheena Ramdeen, Jiling Chou, Sarah J Norman, Sarah H Elgendi, Adrian Llama, Farah I Kitana

**Affiliations:** Medstar Washington Hospital Center/ Ascension Saint Agnes Hospital, Washington, District of Columbia; MedStar Washington Hospital Center, Washington, District of Columbia; Medstar Health Research Institute, Rockville, Maryland; MedStar Washington Hospital Center, Washington, District of Columbia; Medstar Washington Hospital Center, Laurel, Maryland; MedStar Washington Hospital Center, Washington, District of Columbia; Medstar, Silver spring, Maryland

## Abstract

**Background:**

Therapeutic drug monitoring (TDM) of vancomycin is a fundamental part of optimizing efficacy and adverse effects. The Infectious Diseases Society of America (IDSA) consensus guidelines recently recommended a change in vancomycin monitoring from trough-based levels to area under the curve/minimum inhibitory concentration (AUC/MIC). The objective of this study was to compare the incidence of acute kidney injury (AKI) utilizing trough vs AUC/MIC vancomycin monitoring.

**Methods:**

This retrospective cohort study included patients ≥18 years old that had received vancomycin for >48 hours. Patients who developed an AKI within 48 hours of vancomycin were excluded. One cohort included patients monitored by vancomycin trough levels from January to June 2019, and the second cohort included patients monitored by AUC/MIC from July to December 2021. The primary outcome was incidence of AKI per Kidney Disease Improving Global Outcomes (KDIGO) definition. Data were analyzed using a multivariate logistic regression model with covariates that are described to be associated with AKI.

**Results:**

A total of 371 patients were included; 229 patients from the trough-based cohort and 142 patients from the AUC/MIC cohort. The incidence of AKI was 20.1% in the trough-based cohort and 13.5% in the AUC/MIC cohort (p=0.153). Around 39% and 30% patients were admitted to the intensive care unit from the trough based cohort and the AUC/MIC respectively. A multivariate logistic regression analysis identified higher odds of AKI among patients with supratherapeutic vancomycin levels regardless of dosing method, OR=5.89 (95% CI 3.03-11.54) ,and the OR comparing trough based dosing with AUC/MIC dosing was OR=1.1 (95% CI 0.58-2.12).

**Conclusion:**

The incidence of AKI was higher in the vancomycin trough-based cohort vs AUC/MIC cohort. The difference was not statistically significant, but these findings are clinically relevant to practice. These findings align with the IDSA guidelines and suggest that vancomycin AUC/MIC monitoring may cause less nephrotoxicity.

**Disclosures:**

**All Authors**: No reported disclosures.

